# Smart Wireless Sensor Technology for Healthcare Monitoring System Using Cognitive Radio Networks

**DOI:** 10.3390/s23136104

**Published:** 2023-07-02

**Authors:** Tallat Jabeen, Ishrat Jabeen, Humaira Ashraf, Ata Ullah, N. Z. Jhanjhi, Rania M. Ghoniem, Sayan Kumar Ray

**Affiliations:** 1 Faculty of Engineering and Information Technology, University of Technology Sydney (UTS), Sydney 2007, Australia; tallat.jabeen@student.uts.edu.au; 2 School of Interdisciplinary Engineering & Sciences (SINES) NUST, Islamabad 44000, Pakistan; ishrat.jabeen@sines.nust.edu.pk; 3 Department of Computer Science and Software Engineering, International Islamic University, Islamabad 44000, Pakistan; humaira.ashraf@iiu.edu.pk; 4 Department of Computer Science, National University of Modern Languages (NUML), Islamabad 44000, Pakistan; aullah@numl.edu.pk; 5 School of Computer Science SCS, Taylor’s University, Subang Jaya 47500, Malaysia; sayan.ray@taylors.edu.my; 6 Department of Information Technology, College of Computer and Information Sciences, Princess Nourah bint Abdulrahman University, P.O. Box 84428, Riyadh 11671, Saudi Arabia; rmghoniem@pnu.edu.sa

**Keywords:** smart sensor, sensing system, wearable sensor, health monitoring, encryption

## Abstract

Programmable Object Interfaces are increasingly intriguing researchers because of their broader applications, especially in the medical field. In a Wireless Body Area Network (WBAN), for example, patients’ health can be monitored using clinical nano sensors. Exchanging such sensitive data requires a high level of security and protection against attacks. To that end, the literature is rich with security schemes that include the advanced encryption standard, secure hashing algorithm, and digital signatures that aim to secure the data exchange. However, such schemes elevate the time complexity, rendering the data transmission slower. Cognitive radio technology with a medical body area network system involves communication links between WBAN gateways, server and nano sensors, which renders the entire system vulnerable to security attacks. In this paper, a novel DNA-based encryption technique is proposed to secure medical data sharing between sensing devices and central repositories. It has less computational time throughout authentication, encryption, and decryption. Our analysis of experimental attack scenarios shows that our technique is better than its counterparts.

## 1. Introduction

The Internet of Things (IoT) supports an ecosystem in which digital devices and computing sensors can establish human-to-human or human-to-computer interaction. Wearable computing Nano Sensors (NSs) are used in Wireless Body Area Networks (WBAN) to gather health-related data from the patient’s body and transfer them to a biomedical server. This kind of communication demands a high level of security measures to guard against attacks by intruders [1]. The DNA Encryption Algorithm (DEA) [2] is one such security measure. Authentication is mandatory to protect the medical networks from untrusted users [3]. DEA is adopted to protect data transmitted between nano sensors and the biomedical server through the Cognitive Radio Network (CRN) [4]. The CRN optimizes radio resource usage by exploiting the unused channels with an appropriate impedance moderation procedure. It first detects the idle spectrum from multiple base stations and then transmits data to the hospital’s server via a gateway. Depending on their priority, primary and secondary users are granted access to this spectrum [4].

Secure access is mandatory to prevent intruders from undertaking malicious activities. Secure communication demands that messages sent between communicating nodes be encrypted and that the network hold a key for both encryption and decryption. In this article, the ElGamal key generation algorithm and the lightweight encryption and decryption process are used to generate smooth data transmission. Data is converted into ciphertext using a DEA encryption algorithm. The data will be transformed by an algorithm using the encryption key in a predictable manner such that the encrypted data looks random; it can be decrypted and returned to plaintext using the decryption key. Protecting the secrecy of data collected by sensors or transferred over the internet or any other computer network is the main goal of DEA encryption. The requirement to adhere to compliance rules frequently drives the implementation of DEA encryption in addition to security concerns.

IoT-based real-time health-monitoring systems assist the doctors and nurses who can administer the quickest and most accurate medical care with real-time access to patients’ medical information and test results. This fact motivated us to work in this domain. The implementation of security algorithms and authentication processes can reduce the risk of illegal access to healthcare. A real concern with the WBAN-CRN system is its vulnerability to security attacks and excessive power consumption, which may make the system unreliable. Attackers can manipulate data through fake injection and can cause interference by maliciously congesting the traffic. 

In this work, we introduce a new encryption technique that is based on the DEA algorithm and uses the ElGamal algorithm to generate the encryption key. The proposed scheme focuses on the IoT model’s authentication of the patients and sensors using the CRN that exploits idle bandwidth to route data traffic. 

Our contributions to this research field are as follows:

(1) We propose a DNA-based encryption technique to secure medical data sharing between sensing devices and central repositories.
(2) Our proposed technique combines a delicate encryption algorithm, namely the DNA-based Encryption Algorithm (DEA), with a good key generator algorithm to secure the WBAN-generated data within the CRN.
(3) Our proposed technique has less computational time throughout authentication, encryption, and decryption.
(4) The authentication process and encrypted data ensure that only valid users gain access to the network system with our proposed technique.
(5) Our analysis of experimental attack scenarios shows that our technique is better than its counterparts.

The remainder of the article is structured as follows: Section 2 reviews the current security techniques in the literature. Section 3 details our new approach. Section 4 presents our evaluation and results; Section 5 concludes the work.

### Literature Review

The wide spread of IoTs has reached numerous domains, including healthcare. As a result of its expansion, people are more vulnerable to security and privacy breaches [3]. This article proposes a secure agenda based on low power and limited resources. The proposed system uses the AES-CTR mode to initialize a counter value, which is then incremented over a pseudo-random sequence using a variable named (IV) initial vector value. The size of the counter value for encryption depends upon encryption algorithms such as AES-128 bits; this size is also 128 bits. It uses a simple XOR operation as CTR mode used an XOR operation. We also use a similar approach in our proposed work. The design involves the intra and inter WBAN [4] approaches. In the former approach, the sensors around the patient’s body utilize a personal server as a passage to transmit a signal towards the next level. In the latter approach, the patient’s basic healthcare information is recovered by interacting with the primary system via the web. Beyond WBAN, there is another vital basic phase when the medicinal condition database or biomedical server is set up. Because it is made up of incredibly basic materials, security and protection are the most important considerations. To validate the patient’s information, many information security solutions are provided. To validate the information, it uses an elliptic bend cryptography (ECC) technique with a solid key management structure. To ensure the information’s unwavering quality and security, the system uses registration, verification, and key exchange procedures. 

Different encryption and decoding strategies are proposed for the information security in the WBAN domain, yet at the same time there is a risk of multiple attacks. The malicious node attack in IoT-enabled WBAN is identified and dealt with using the BAN trust strategy [5]. It is difficult for a sensor node to connect legitimately, but it is necessary to transmit data. To determine whether a node is trustworthy to communicate or not, one must confirm whether it has ever interacted with another node; supposing it has, the recommendations it receives from others are critical in determining the trustworthiness of that unknown node. In [6], a genetic-based algorithm for data security is used to protect against attacks. Multiple schemes are analyzed for data security. The comparison analysis of lightweight numerically encrypted data with the optimal protocol is the best system for data delivery overall. 

There are two kinds of cognitive radio-based systems: three-tiered and centralized cognitive networks. The former approach comprises of intra, inter and beyond WBAN communication. Three systems are included in the Cognitive Radio system: inventory system, CR controller, and CR client. The inventory device preserves NS information and passes it to the CR controller. CR controllers control the power of CR clients. The intra-based approach involves NSs placed in or above the body which interconnect with a cognitive radio controller within a range of two meters [7,8]. The CRN can be merged with IoT as CRIoT to solve the scarcity problem in IoT. In [9], a packet-sharing mechanism is presented for CRIoT to reduce the delay and the number of dropped packets. Our work also reduces the delay inherently due to the CRIoT model. In [10], a trust-aware packet routing mechanism is presented for social CRIoT. It uses a game theoretic-based approach for trusted channel selection. Our work also ensures secure communication by presenting a cryptography function for CRIoT that is also applicable to social scenarios. M. Shafiq et al. presented routing mechanisms that are based on location protocols, data-centric protocols, hierarchical protocols, mobility protocols, protocols based on multipaths, protocols based on heterogeneity, and protocols based on QoS [10]. Similarly, our work also adopts the CRN-based routing mechanism for better communication and fewer delays. Khalid et al. presented the half-duplex-based routing mechanism for the CRIoT to improve the throughput [11]. 

**Figure 1:**
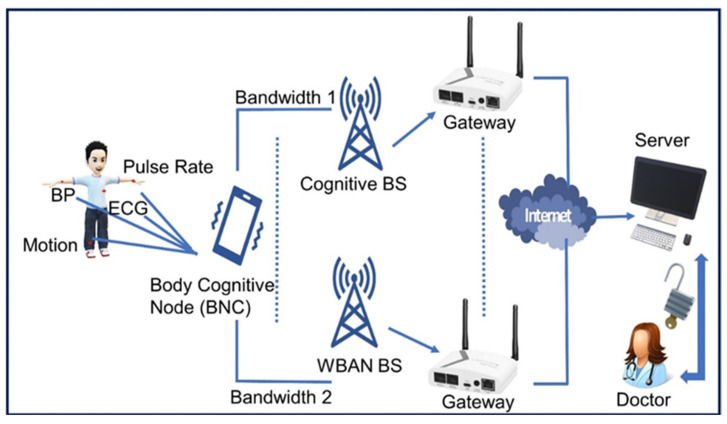
The WBAN-CR Network Architecture.

In CRIoT, the consumer senses the bandwidth to recognize the presence of idle spectrum with any frequency or time domain [12]. Moreover, a simulator for the full-duplex radio networks over IoT is presented in [13], where packets are sent and received among the sensing devices. Primary customers share their data arrangement or possibly code book with the secondary transmitter in overlay. Consider a scenario where the information for medicine and hospital observation data begins from the facilitator. In this case, the two applications have side data on each other’s messages; on a similar frequency band, the overlay CR sends the MWBAN information to two targets. The primary patient is the medicine transmitter, and in-clinic knowledge is the secondary patient. The system architecture of WBAN over CRN intends to transfer a limited amount of sensed data towards the remote server through the closest base station of cognitive networks [13]. Figure 1 illustrates the Body Cognitive Node (BNC). BNC is the main part acting as a gateway between WBAN and CRN. It transmits data from sensors to the server. Data can be transmitted layer wise through CRN [14]. 

A CRN moderates interference and enhances the effectiveness of the limited spectrum usage. In order to prioritize the access of the frequency channel, there are two types of users: primary and secondary. Primary users are favored compared to secondary users because of real-time and crucial data transmissions and because they have higher priority in choosing the available bandwidth. Spectrum sensing now requires sensing the idle bandwidth information to route the data, and the next step is to access the bandwidth. Bandwidths access functions coordinate to sense the idle spectrum and update the WBAN’s channel access time. The amount of radio spectrum required for applications, such as mobile telephony, digital video broadcasting (DVB), wireless local area networks (Wi-Fi), wireless sensor networks (ZigBee), and the Internet of things [15,16], is immense and continues to rise. By 2021, nearly 50 billion cellular devices will be linked, necessitating a significant amount of spectrum [17]. The CR- based paradigm is explored for a routing mechanism in [18], whereas the contention window-based mechanism is explored in [19]. In [20], a channel-hopping mechanism is designed where secondary users can avail the network without clock synchronization at the global level. It improves throughput and average time. The main benefit is that it provides wireless devices with opportunistic access to more bandwidth, allowing them to boost their achieved throughput [21]. Intrusion detection is the method of detecting harmful or illegal computer or network activities in CRNs [22]. Better use of existing spectrum resources has been accomplished with the aid of CRNs [23] by leveraging underutilized licensed spectrum [24,25]. For automation applications based on wireless communication, cognitive radio considerably reduces spectrum scarcity [26]. Secondary users (SU) in mobile cognitive ad hoc networks regularly detect the behavior of main users (PU) and opportunistically utilize PU’s idle licensed channels [27]. It is critical to develop viable techniques to deal with interference, and to make efficient use of temporarily accessible frequency bands in order to maintain a high degree of communication quality [28,29]. It is also observed that various related schemes in the literature provide high computational complexities in terms of deployment. These complexities enhance the cost of the whole system, which renders the structure difficult to maintain. The best resource distribution for cooperative CRN with opportunistic licensed spectrum access is examined. CRN is used as a protocol to forward encrypted data towards the server. Therefore, CRN is an optimal and cost-effective selection as a protocol for data delivery. 

Table 1 presents a comparison analysis of multiple related schemes, with the proposed scheme based on some security attack parameters. Literature-related security techniques had some stumbling blocks with attacks, but the proposed security scheme provides all the safety parameters mentioned in Table 1. Some solutions described in the related articles protect against attacks denoted by the rightly rectified symbol. 

Therefore, it is observed that a proposed DNA-based scheme with an authentication process is lightweight and efficient in terms of security parameters and time complexity. Ismail et al. [34] and Zhao et al. [35] provide DNA-based techniques to secure data over the networks. Technology, commerce, and social conventions have all seen rapid advancements in the twenty-first century [36]. Comparison analysis with the proposed scheme and the literature scheme is provided in Section 5. 

## 2. Proposed Methodology

The architecture of our WBAN-CRN system comprises the DNA-based Security Encoding Algorithm (DEA) along with the CRN protocol to encrypt data and authenticate patients and devices. The DEA is used to encode the data collected by the IoT-enabled WBAN system, and the ElGamal algorithm is used to generate the encryption key. The ciphertext is then transmitted wirelessly to the biomedical server through the CRN routing. The WBAN consists of nano sensor nodes attached to the patient’s body to monitor and collect vital health data, such as body temperature, sugar level, heart beats (ECG), blood pressure, etc. This data is routed over the network through the CRN. It is used with multiple electromagnetic spectrums to transfer data to the server. It detects the idle spectrum and updates the WBAN channel sensing. The transmission of data and allocation of the idle spectrum are also observed in the cognitive network. Figure 1 presents the architecture of the WBAN-CR network, which consists of four components: sensor BNC nodes, access points, gateways, and the medical server that is easily accessible to physicians. Each transmission between these components consumes less time and fewer computational resources. 

The first lines of defense against impersonation are the authentication of patients and the sensor devices attached to them. 

The patients use their unique IDs associated with cellular phones to register with nano sensors that use the device IDs to register with gateway. The nano sensors collect data and transmit them to the gateway for routing. In the sensor registration phase, nano sensors share unique identity SID for registration in the gateway’s database. 

The proposed DNA Encryption Algorithm (DEA) demands less computational time and memory space. First, an encryption key is generated using the ElGamal algorithm, and this key is subsequently used to encrypt data using the DEA algorithm. A symmetric encryption algorithm uses one key to encrypt and decrypt data while an asymmetric algorithm uses a private key to encrypt data and a public key to decrypt them. The proposed scheme uses the ElGamal algorithm to produce a symmetric key to generate a mutual password for data sharing. First, two common prime numbers, P and D, are decided by the parties who wish to establish communication. Then, T is used to generate the ciphertext, where T=(E)^d mod P, and E is a selected value. 

**Figure 2:**
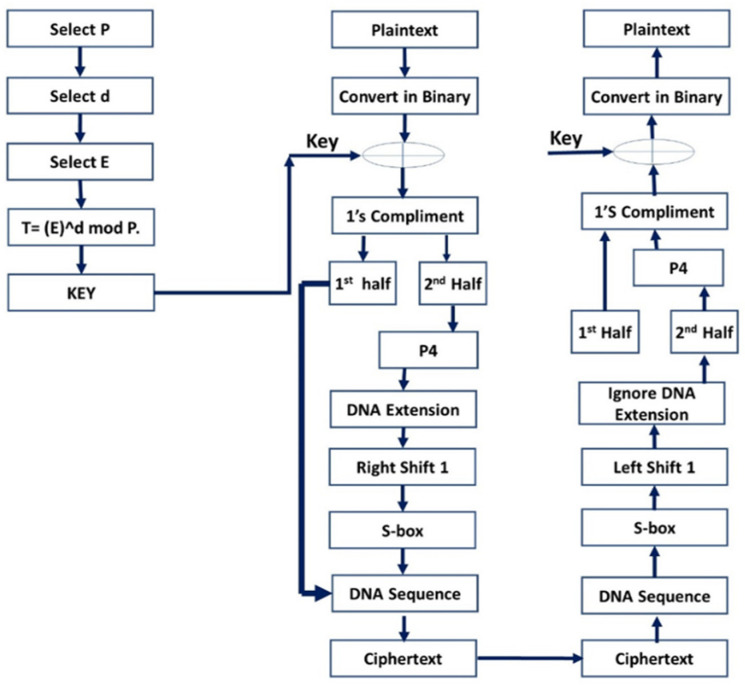
Encryption and Decryption Algorithm.

Figure 2 elucidates our encryption scheme. In the decryption phase, the ciphertext is converted into binary from a DNA table that is DNA sequences. The data obtained are used to recover values from the S-box to apply the circular left shift one. Next, four bits of two subsets each are produced, and the first half of the four bits are taken to perform the operation while ignoring the second half of four bits (as they were extended from the DNA). 

Then, P4 substitution is applied on the first half of the four bits, which combines the previous bits to form eight bits. Now, one’s compliment is taken, and XOR operation is performed with the key generated by the ElGamal algorithm.

The resulting binary data is eventually presented and displayed to the patient in decimal format (e.g., ECG data. The P4 is used to swap the four bits’ binaries with each other on the corresponding value in the table. This operation is performed on the second half of the encryption algorithm bits. There are four indices, 1, 2, 3, and 4, to replace the corresponding indices. For example, 1 is replaced by 4, 2 is replaced by 1, 3 is replaced by 2, and, lastly, 4 is replaced by 3. The S-box describes the workings of the proposed algorithm in steps. These steps are discussed in detail. The simple S-box contains binary values which are substituted in the encryption algorithm on demand and used while decrypting the data. The simple S-box contains the row of 0, 1 and the column of 0, 1. Further, 00 in the box results into 1 and 01 yields 0; likewise, 10 results in 1 and 11 yields 0. DNA standard contains binary values of two bits each and a corresponding DNA sequence. There are four rows of 11, 01, 10, and 00, with their corresponding DNA sequences. Moreover, 11 comprises the sequence ‘G’ and 01 contains ‘A’; likewise, 10 has a sequence of ‘T’. Lastly, 00 provides a DNA sequence of ‘C’. In an encryption algorithm, the first binary values are used to extend the bits. Next, the AND operation of the first row 11 with third 10, second 01, and fourth 00 are performed. The obtained group of four bits is used in encryption for further action. At last, the binary value converts DNA sequence into cipher text.

NS detects WBAN’s data and gives information to a personal device (PD) that sends data over the idle bandwidth spectrum, which is then linked to BS. If PD receives data from the NSs securely, then the idle spectrum is sensed for the transmission of data. If PD does not receive encrypted data from the NS, then the idle spectrum of bandwidth is not sensed. Data is sent towards BS of cognitive networks successfully if it receives data from PD, as illustrated in steps (2) to (13). BS transfers encrypted data towards the gateway securely using DEA. If BS receive data from the bandwidth securely, then the gateway transfers through clouds. If BS is not receiving data from the bandwidth securely, then the gateway does not transfer any data for further actions. But if data is sent from BS to the gateway successfully, data is transferred to the medical server, as demonstrated in steps (18) to (31) (Algorithm 1).
**Algorithm 1:** Key Generation and DNA Encryption.***Key Generation***  ***Begin***
  *Select Prime number P.*  *Select Private Key D.*  *Select Public Key E.*  *T = (E)^d mod P.*   *Value of T will be key value*   ***End******DNA based Encryption (at WBAN’s Sensors)***8. ***Begin***
9. ***for*** *each NS ∈ PD **do***10. *Sensor node sends authenticated data towards PD through DNA Encryption securely*11. *Then idle spectrum is being sensed to transfer data*12. *Through idle bandwidth spectrum PD transfer data to BS*13. ***end for***
14. ***for*** *each NS ∈ PD **do***15. ***if*** *(PD receives data from NS securely) **then***16. *encrypted data is ready to transfer over idle bandwidth spectrum*17. ***else if*** *(PD does not receive data in given slot from NS) **then***18. *Bandwidth is not sensed for idle spectrum*19. ***else if*** *(BS receives data from PD successfully) **then***20. *data is transferred to cognitive networks BS*21. ***end if***
22. ***end for***
***DNA Encryption Algorithm (DEA) (at Cognitive networks)*****Begin**23. *BS transmits data to gateway safely encrypted using DEA*24. *Data is transferred from the gateway to the medical server.*25. *Data is detected by medical servers for further action.*26. *for each BS GW do*27. *If (BS safely receives data from the spectrum), then*28. *Gateway is used to transport data over clouds.*29. *Data is not identified by the Gateway to send over clouds if (BS does not receive data from any spectrum in specified slot).*30. *End if*31. *If (data from BS to Gateway) is true, then*32. *Gateway clouds deliver data to the medical server.*33. *End if*34. *End for*

### 2.1. Mathematical Modelling

In Y=x⨁k, Y is provisional text which is updated according to the operations. Plaintext X calculates XOR with key k. Next, Y1=Yc, where Yc=YC1.YC2. In the equation, Yc is calculating one’s complement of the generated short-term cipher, which is equal to Y1. Next, Yc is divided into two halves, denoted by YC1 and YC2. In the case of Yc=YC2→P4, P4 is applied over the second half of the complement YC2 of four bits. P4 is the permutation which substitutes bits accordingly. Y2=P4∼TDNA and Y3=≫Y2. Now, four bits of permutation P4 need to extend into the eight bits with the help of the DNA table, which is equal to Y2. Then, eight bits converted Y2 is circulated one shift right, which creates the temporary cipher of Y3. In Y4=Y3→S−box and Y5=YC1 U Y4. The S-box is applied over the right-shifted bits of Y3, which again convert eight bits into four bits. This short-term cipher is equal to Y4. Now, the previous first half of the complement YC1 is associated with Y4 to convert into eight bits again, which generates the cipher of Y5 i.e., Y5=DNAsq and Z=Y5. The DNA sequence is applied to Y5 from the DNA table, which is equal to Z and is actual generated ciphertext.

### 2.2. Cognitive Radio Network Protocol

The sender node selects ‘A = (1 − n, n = prime number)’ and the receiver node selects ‘B = (1 − n, n = prime number)’. The sender node selects three random numbers, ‘P’,‘D’, and ‘E’, and then calculates the value of T, which will be used to generate the key. The sender node calculates the T value from the formula T=(E)^dMOD P, which is the key used for encryption. The sender node chooses plaintext to be sent to the sender node, instead of converting the plaintext to a binary value. If the key is generated, then the sender node uses the generated key and the binary value of the XOR key. One’s complement of the temporary cipher text is taken. Then, the generated text is divided into two subsets, and subset 1 is left as it is and the operation is performed on subset 2. Parameter 4 (P4) is applied to subset 2. Now, the four bits are extended from the DNA extension to make 8 bits of data. The circular right shift 1 is applied to eight bits. The values are substituted from the S-box, which again forms four bits of data. Now, subset 1 is added to the generated four bits of data to form eight bits of data. The sender replaces the DNA sequence from the DNA table that is actually the ciphertext received. Otherwise (if the key is not produced, then the key is first generated, then repeated), the sender node submits the encrypted text and sender node ID to the bandwidth sender node. After receiving the encrypted text from the receiver node, it then performs the operation again but in reverse.

**Figure 3:**
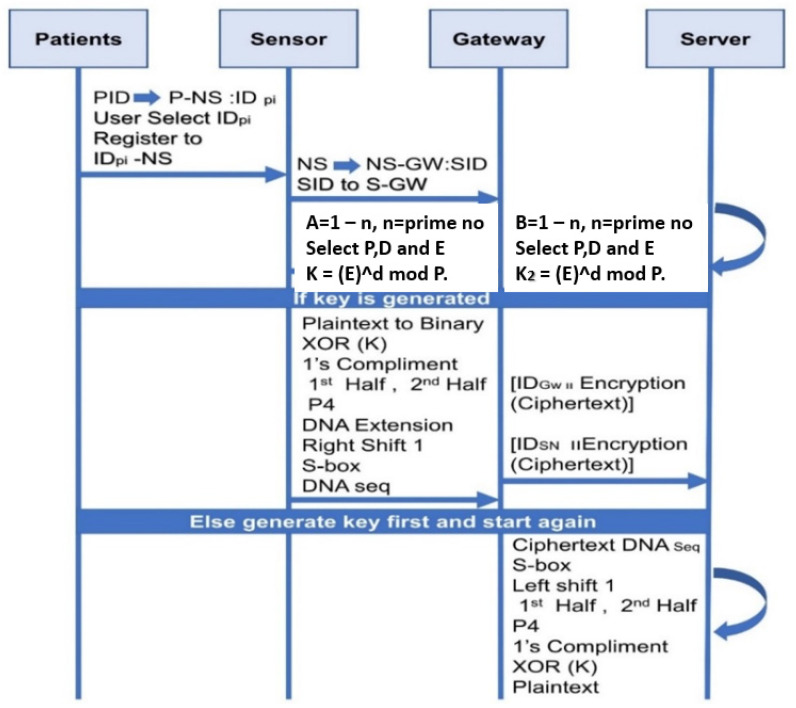
Secured Routing Protocol in a Cognitive Radio Network.

Figure 3 demonstrates that Steps (1)–(6) indicate that WBNS’s sensor-generated plaintext is transferred to the server through the cognitive network. The plaintext of WBAN is converted into 8-bit binary. The 8-bit binary values of step 2 are then XORed with the same bit of key generated by the ElGamal key generation algorithm. Then, one’s compliment of the resulting binary data generated in step 3 is taken. Next, the generated 8-bit binaries are divided into two halves. In Steps (7)–(12), initially it leaves the first half as is and considers only the second half of the data. Parameter 4 (P4) is applied on the second half, which generates four bits of data with their corresponding indexes.

Next, the four bits in step 7 are extended to step 8 by inserting the DNA values and performing the AND operation on the first value with the third value and on the second value with the fourth value from the DNA. After that, a circular right shift is performed on the 8-bit-generated data from step 8. The corresponding bits from the S-box which convert binary data back into 4 bits are substituted. Next, the previously ignored bits of the first half in step 6 which make 8-bit binary data are added. Finally, the 8-bit binary data is substituted into the DNA sequence given in the DNA table. Step 13 shows the DNA sequence for the ciphertext of the WBAN data.

The receiver receives encrypted data that is not readable at step (28). First, the DNA sequence of the ciphertext is substituted with their corresponding binaries. The S-box is applied to the generated values of step 2. A circular left shift is performed on the 8-bit data. The 8 bits of data is divided into two halves, which form two subsets of 4 bits. The second half of the bits in step 5 are ignored, as they are DNA-extended bits. Now, parameter 4 (P4) is applied on the first half of these bits and combined with the previously assigned bits—which forms 8 bits of data. One’s compliment of the resulting 8-bit data is taken. Now, the XORed operation is performed with the ElGamal key generation algorithm. The value is then translated into ASCII decimal places after all the decryptions. The SDEA is a secure algorithm that has a low computation time and a good response and renders it difficult for attackers to intercept plaintexts.

For the duration of the enhanced network, the deployed sensor node needs judicious use of energy sources. The forwarder node relays information from all sensor nodes to the sink. Sensor nodes choose a path to the sink node that is idle and consumes less energy. The proposed work centered on determining the best data transmission path and lightweight security algorithm to enhance its performance efficiency. The proposed energy-aware routing protocol lowers the overall network implementation cost and the amount of energy consumed by the network and sensors.

## 3. Security Analysis

Security analysis is mandatory to secure the data from different attacks. Network-routing data may be compromised by an attacker, and therefore data transmission must be secure. The use of wireless networks and sensor devices makes the network vulnerable to multiple types of attacks, such as plaintext attacks, eavesdropping attacks, tempering attacks, jamming attacks, sybil attacks, and collision attacks. Secure wireless networks are essential for preventing unauthorized access to data by implementing these novel techniques.

### 3.1. Plaintext Attack

**Figure 4:**
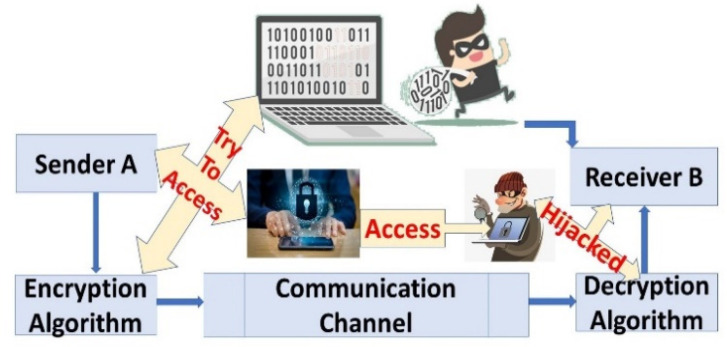
Plaintext Attack.

After obtaining the plaintext and ciphertext, the attacker may attempt to analyze the relationship between the plaintext and ciphertext. In cryptography, this is a very basic attack. The attack scenario is where the user sends the data for encryption and the piece of plaintext is encapsulated by the attacker, as shown in Figure 4. With this known piece of data, the attacker attempts to recover the encryption algorithm that is then used as P(C(S,R)=Z(P,C) for the decryption process, where P is plaintext that transmits data with a ciphertext pair from A to B, and Z is the attacker that attempts to recover data with known plaintext. Our introduced work prevents plaintext attacks as the users do not send simple plaintext over the network. A known plaintext attack necessitates the recovery and analysis of a matched plaintext and ciphertext pair. The purpose is to determine which key was used. If the attacker recovers the key, then the attacker can decode other ciphertexts encrypted with the same key. Plaintexts always use the El Gamal key generator that uses randomly chosen parameters, which are extremely hard for the attackers to discover.

### 3.2. Eavesdropping Attack

A three-person scheme, including a sender, receiver and a middleman (the attacker), is the proposed system. The smooth transmission channel is often disrupted by middleman, and the conversation between sender A and receiver B is secretly listened to by intruder Z as E(A,B)=Z(E(A,B))+Z(Ei+1(A,B)), where E is the encrypted data and Ei+1 shows the addition of a spoofed packet in the encrypted data flow. The interaction is eventually manipulated. Due to the DEA algorithm and the authentication method, this scenario could be evaded in the proposed framework. The authentication process prevents third parties from intercepting the conversation.

### 3.3. Tempering Attack

Tempering is a sort of attack in which an attacker gains physical access to a node or obtains sensitive data on the node, such as cryptographic secret keys or other confidential data. The attacker, who could change the system, then constrains it or substitutes communication. Outsider Z gains physical access to the data flow by breaking communication between nodes A and B as $\left(NA,NB)=(Z(NA)),(Z\left(NB\right)\right)$. This type of attack can only be guarded against if the nodes are placed in physically secure places. In our proposed solution, we do not consider the physical security of the nodes.

### 3.4. Jamming Attack

**Figure 5:**
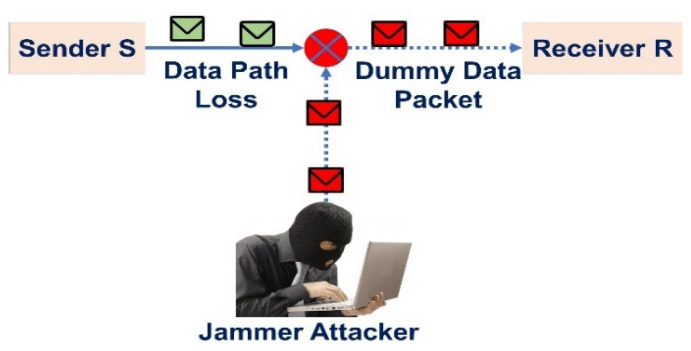
Jamming Attack.

Jamming is an attack which interferes with the frequencies of the radio that the nodes of a system are using. It is a subset of denial of service (DoS) attacks. The data transmission path between the sender and receiver is dropped because of a jamming attack. The attacker constructs an alternative communication path with the receiver, as presented in Figure 5. The data transmission path between sender S to receiver R is dropped and the jamming attacker Z links a new path to transmit dummy data packets to receiver R as (NS,NR)=(NS),(Z(NR)). Our proposed technique prevents this attack due to CRN. A cognitive network uses a priority-based and idle bandwidth spectrum for data transfer. The data transmission path is not obstructed by this idea. Data collision has also been prevented due to the adaptation of the idle spectrum.

### 3.5. Sybil Attack

Sybil attack is a type of attack that obstructs the routing protocol using false identities. Data transmission between the original node and the authenticated node is disrupted by the Sybil nodes that are using their multiple fake identities to create hindrances to communication. The proposed technique avoids the Sybil attack by using the authentication of the involved nodes and other stockholders in the networks. Therefore, unauthenticated Sybil nodes are not considered part of the system, and transmission remains smooth. It involves rounds of communication between original and authenticated nodes with the Sybil barriers. In the equation (NO,NA)=(NO),(NS(NA)), NO is message-originated node that transmits data towards the NA node, and NS is the Sybil attacker that affects the smooth data transmission.

### 3.6. Collision Attack

**Figure 6:**
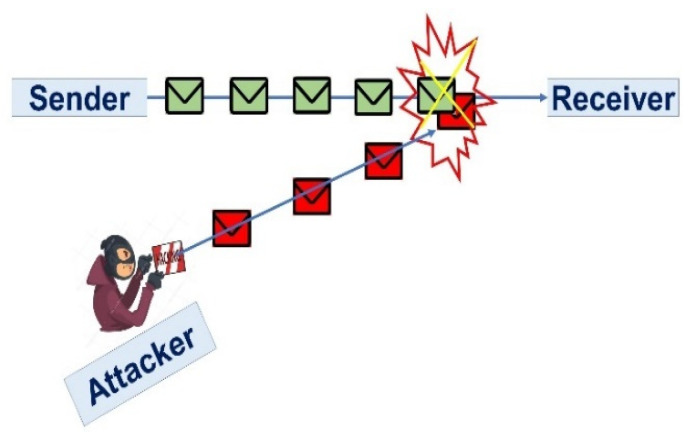
Collision Attack.

When more than one node tries to forward data, then a collision of data occurs. In this situation, an attacker may intentionally cause a collision to discard the transmitted data. An example of the concept of data collision is in attacker Z sending fake data to hit the original data packet. In the equation, (NS,NR)=((NS)×(Z))(NR), NS is sender node and NR represents the receiver. The proposed DEA technique prevented this attack by using authentication and a cognitive radio protocol, as shown in Figure 6.

## 4. Results and Analysis

In our experiments, we used the MATLAB 2013a to simulate the cognitive radio networks. The field space of the simulation was 100 × 100 m. The body sensors collected ECG data. WBAN detected the ECG of the human heart and provided detailed data results. CRN was used as a routing protocol to transfer the WBAN’s data to the biomedical server using a bandwidth spectrum. The CRNs sense the idle spectrum in multiple antennas through an improved energy detector. This simulation provides detailed results of the proposed system. There are two types of evaluation measures: performance evaluations and results evaluations. Performance evaluations measure the time taken by the algorithm to encrypt or decrypt the data. We compared the computational times, which are the times calculated by the algorithm to complete specific tasks in limited time slots. The base schemes, AES-CTR [37] and ECC [38], are compared with ours.

### 4.1. Analysis of Authentication Time Complexity

**Figure 7:**
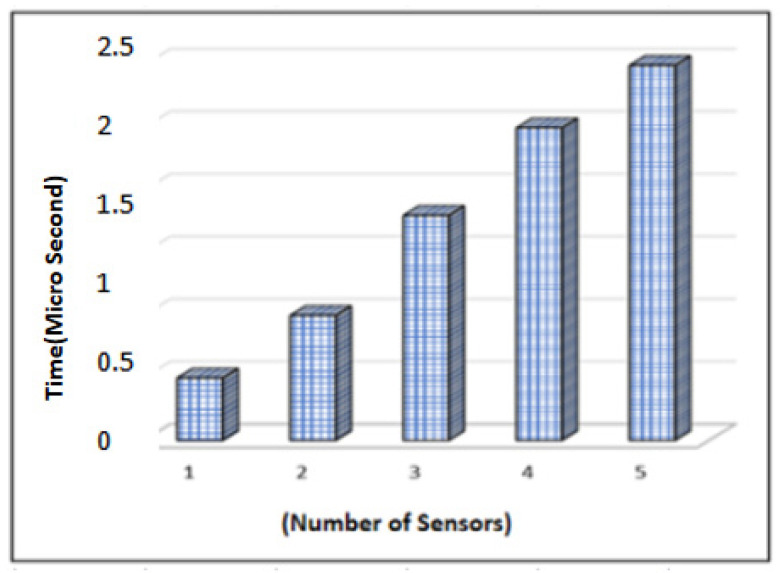
Time Complexity of Authentication Process.

The computational time interval of sensors’ authentication of patients and gateways is observed. It is noted that as the time to register tiny sensors increased, the response time also increased—e.g., when sensor1 registers in 0.5 microseconds then the 5 micro sensors take about 3 microseconds. Figure 7 illustrates that the overall time taken to authenticate people is slower and efficient.

### 4.2. Time Complexity for ElGamal Key Generation

**Figure 8:**
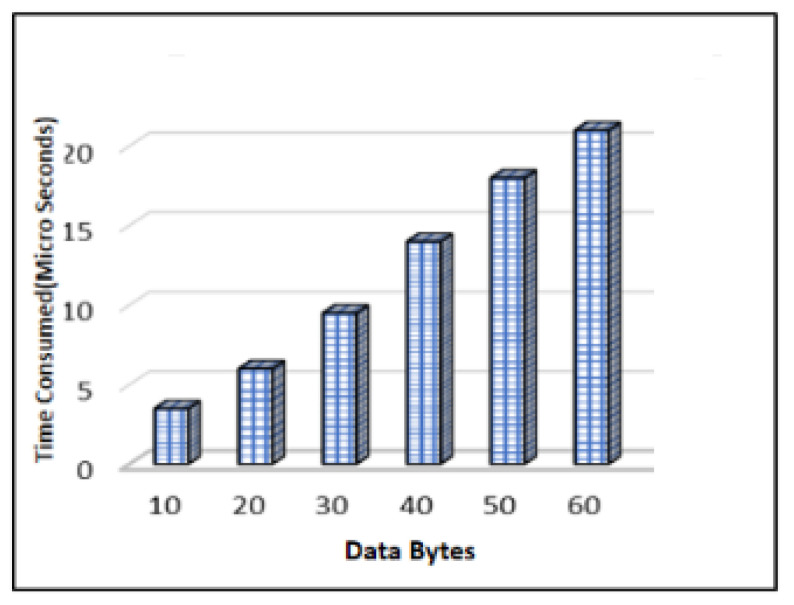
Time Complexity of ElGamal Key Generation.

The computational time is the time it takes the ElGamal technique to produce a key. The data bytes and time taken by the method to create the key for the encryption procedure are shown in Figure 8. The time used by the procedure increases as the number of data bytes increases. For 30 bytes, it takes 9.5 microseconds to compute the data bytes for key production, whereas for 60 bytes the same operation takes 21 microseconds.

### 4.3. Time Complexity Comparison for Key Algorithms

**Figure 9:**
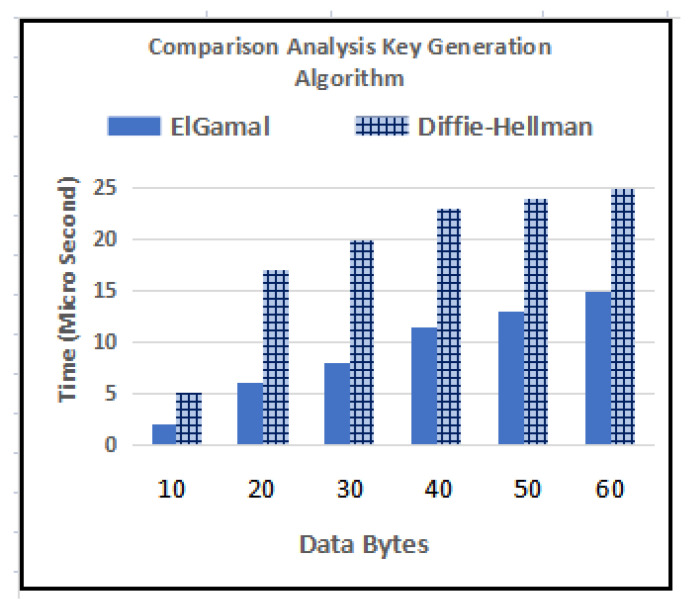
Time Complexity Comparison for Key Generation.

The ElGamal key generation algorithm is used to support the proposed encryption scheme, and its comparison analysis is performed with the Diffie–Hellman Key generation method [38]. In Figure 9, it is observed that as the data bytes increased, the time taken also increased—e.g., one data byte took approximately 2 microseconds in the ElGamal method and the Diffie–Hellman increased to 5 microseconds at same amount of data byte.

### 4.4. Time Complexity for Encryption and Decryption Algorithm

**Figure 10:**
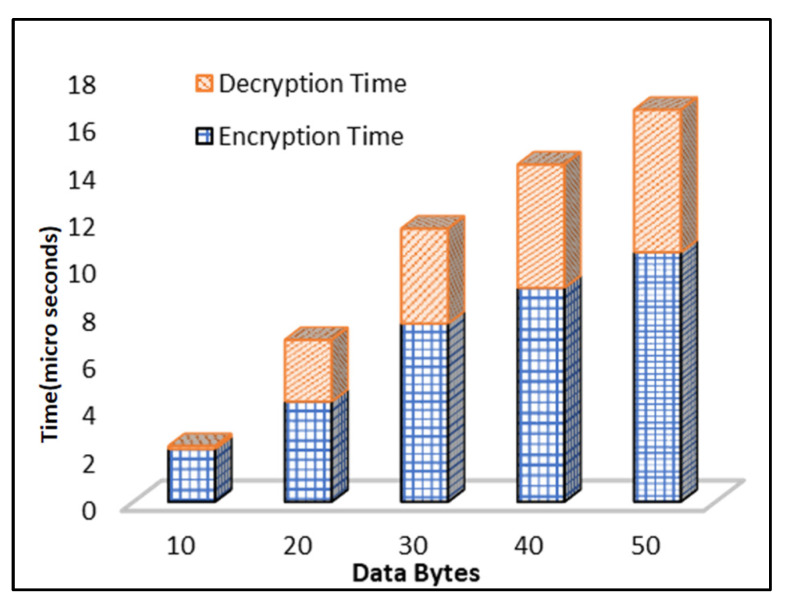
Time Complexity of Encryption and Decryption Algorithm.

Plaintext conversion into ciphertext is known as encryption. Encryption time is the time taken by the algorithm to transform plaintext into ciphertext. The time used by the proposed DEA is estimated. Figure 10 illustrates that time also increases as the data bytes for encryption increase. The proposed DEA requires less time, even with larger data bytes of encryption, as it is an efficient algorithm. It is noted that as the data bytes increase, the time of encryption also increases gradually. In the case of 10 data bytes, the time required is 2 microseconds, while at 30 data bytes it is almost 7 microseconds. Decryption is the process of fetching plaintext from the ciphertext. The time that it takes for an algorithm to fetch the original data is known as the decryption time. Figure 10 elucidates that various bytes of data take different times to decrypt the data. The decryption time for 10 data bytes is 0.5 microseconds, and for 20 data bytes it is approximately 2.5 ms. The time varies according to data bytes.

### 4.5. Time Complexity Analysis for Encryption and Decryption Schemes

**Figure 11:**
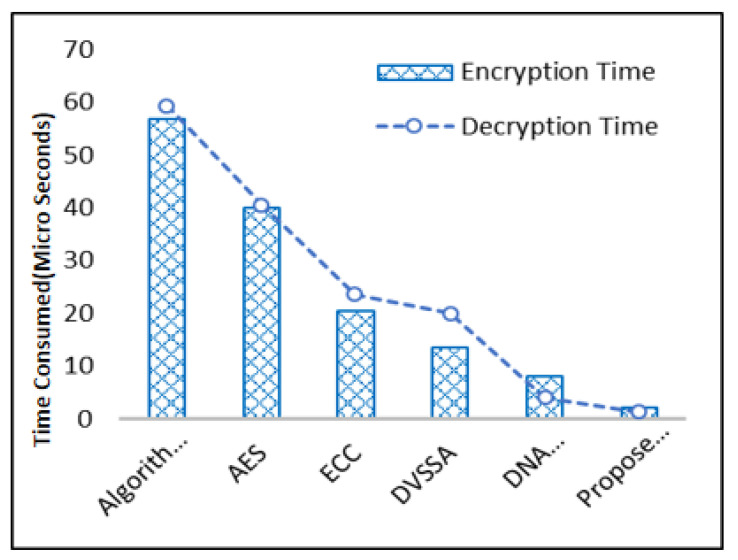
Time Complexity of Multiple Encryption and Decryption Algorithm.

The encoding time of the different schemes is contrasted with the DEA’s proposed algorithm. Compared to the other methods, Figure 11 shows that the data encryption process took less time for the proposed algorithm. For encryption, DEA has less time complexity. Multiple schemes of decryption times are checked because the suggested DEA algorithm has less time complexity compared to other data decryption schemes. The AES-CTR, ECC, and suggested DEA algorithms are timed. AES-CTR takes about 40 microseconds and requires many calculations, whereas DEA takes around 4 to 5 microseconds and requires many computations.

## 5. Discussion

With the high adoption rate of IoT in the medical field, the use of sensors poses many concerns, such as securing their data. Tackling this concern is a challenge due to the limited resources of memory and energy in sensors. Different methodologies have been used to encrypt information, but these methodologies are not considered suitable for remote sensors due to the scarcity of computing resources. In [35], the DNA-based techniques demonstrate data security, and its results are compared with the proposed DNA-based technique, as shown in Figure 11. In [36], DVSSA is used with the DNA-based method and data security and computational energy consumption are plotted in a comparative analysis graph. In [37], various algorithms are used to secure data over the wireless network. It is noted that the proposed DNA scheme required far less computational energy consumption than all other related methods.

We presented an approach that is based on the DEA cryptographic algorithm. To enhance this algorithm and make it more efficient, we used the ElGamal key generator. In CRNs, the routing protocol transmits data by utilizing the bandwidth of an idle spectrum. To secure the detected data transfer, the proposed lightweight approach is implemented on the ECG-detected data. Combined with the CRN protocol, the DNA-based method encrypts and decrypts data. Interference and security attacks are prevented by this methodology. Employing a bandwidth spectrum, CRN is utilized as a routing protocol to send sensing data to the biomedical server. CRNs use a stronger energy detector to detect any unused spectrum via several antennas. Overall, the results collected by the proposed methodology are effective in terms of time complexity and sending data free of attacks and third-party involvement. For the comparison of results strategy, the similarities, differences, and contradictions between the qualitative and quantitative results are identified and explained. The dual axis graphs are used to display insights into two various data points for the comparison of results. It is observed that the results obtained from the proposed technique are better because the technique uses the lightweight and efficient DEA algorithm, whereas the existing algorithm is heavy and difficult to implement and requires more time to implement. Further, the proposed technique uses two axes to easily illustrate the relationships between two variables with different magnitudes and scales of measurement.

## 6. Conclusions

The proposed scheme is applied on the ECG-detected data to ensure secure data sharing. Data are encrypted and decrypted through the DNA-based process along with the CRN protocol. This resolves the problem of interference and security attacks. CRN is used as a routing protocol to transfer the sensing data towards the biomedical server using bandwidth spectrum. CRNs sense idle spectrum in multiple antennas by using an improved energy detector. A secure, DNA-based cryptographic mechanism protects against several security attacks. The CRMBAN system yields approximately 90% better results in terms of complexity, time, and performance; it also reduces power consumption. The system is simulated using MATLAB 2013a. CRN for data routing will be efficient due to smooth data traffic. Experimental results show that no packet loss is observed by avoiding congestion of data traffic. Overall, the calculated results of the system are according to the requirements of the NSs’ capacity.

The proposed approach can be extended using different routing protocols and key approaches. To minimize the computational time, it is imperative to have an algorithm with fewer steps. In future work, we will investigate DNA-based operations using fragmentation-based distributed cryptographic keys.

## Figures and Tables

**Table 1:** Comparison of Security Analysis.

Research Schemes	Plaintext Attack	Eavesdropping Attack	Tempering Attack	Jamming Attack	Collision Attack	Sybil Attack	Selective Forwarding Attack
[30]	×	✓	×	✓	×	×	✓
[31]	×	×	×	✓	×	×	✓
[32]	×	✓	×	×	×	×	×
[33]	×	✓	×	×	×	×	×
**Proposed work**	✓	✓	✓	✓	✓	✓	✓

## Data Availability

Data will be available on request.
